# Genetic Diversity of *Candidatus* Liberibacter asiaticus Based on Two Hypervariable Effector Genes in Thailand

**DOI:** 10.1371/journal.pone.0112968

**Published:** 2014-12-01

**Authors:** Thamrongjet Puttamuk, Lijuan Zhou, Niphone Thaveechai, Shouan Zhang, Cheryl M. Armstrong, Yongping Duan

**Affiliations:** 1 Department of Plant Pathology, Faculty of Agriculture, Kasetsart University, Bangkok, Thailand; 2 U.S. Horticultural Research Laboratory, US Department of Agriculture, Agricultural Research Service, Fort Pierce, Florida, United States of America; 3 Tropical Research and Education Center, University of Florida, Homestead, Florida, United States of America; Shanghai Jiao Tong University, China

## Abstract

Huanglongbing (HLB), also known as citrus greening, is one of the most destructive diseases of citrus worldwide. HLB is associated with three species of ‘*Candidatus* Liberibacter’ with ‘*Ca*. L. asiaticus’ (Las) being the most widely distributed around the world, and the only species detected in Thailand. To understand the genetic diversity of Las bacteria in Thailand, we evaluated two closely-related effector genes, *lasA*_I_ and *lasA*_II_, found within the Las prophages from 239 infected citrus and 55 infected psyllid samples collected from different provinces in Thailand. The results indicated that most of the Las-infected samples collected from Thailand contained at least one prophage sequence with 48.29% containing prophage 1 (FP1), 63.26% containing prophage 2 (FP2), and 19.38% containing both prophages. Interestingly, FP2 was found to be the predominant population in Las-infected citrus samples while Las-infected psyllids contained primarily FP1. The multiple banding patterns that resulted from amplification of *lasA*_I_ imply extensive variation exists within the full and partial repeat sequence while the single band from *lasA*_II_ indicates a low amount of variation within the repeat sequence. Phylogenetic analysis of Las-infected samples from 22 provinces in Thailand suggested that the bacterial pathogen may have been introduced to Thailand from China and the Philippines. This is the first report evaluating the genetic variation of a large population of *Ca*. L. asiaticus infected samples in Thailand using the two effector genes from Las prophage regions.

## Introduction

Citrus huanglongbing (HLB) is the main threat to the citrus industry in Thailand and most of the other citrus producing countries around the world. As one of the leading fruit crops in Thailand, the yield of citrus fruit has significantly declined due to HLB epidemics [1] since this disease first appeared in Thailand in the 1960's [2], [3]. HLB is associated with three Gram negative and phloem-limited bacteria in the genus ‘*Candidatus* Liberibacter’ [4], [5]. *Ca.* L. asiaticus (Las) and *Ca.* L. americanus are transmitted by *Diaphorina citri*, while *Ca.* L. africanus is transmitted by *Trioza erytreae*. Among them Las is the most prevalent species in Asia, South America, and North America. Las infects all cultivated citrus varieties in Thailand and causes various symptoms on citrus plants, mainly on the leaves. Symptom variations on HLB-affected citrus plants were found to be associated with the specific Las populations in Florida based on the genetic diversity found within the Las prophage regions [6]. However, there is very little information regarding the distribution of HLB and the genetic diversity of Las in Thailand.

Prior to the availability of the completed Las genome sequences, the detection, identification and genetic diversity studies of Las bacteria were restricted to the sequences of a few housekeeping genes, such as the 16S rRNA [7], [8], the *omp* gene or the *β*-operon gene loci [9], [10]. The resulting diversity studies using these housekeeping genes revealed limited variation amongst the Las global isolates. Since the publication of the Las genome sequences, a number of loci, especially repetitive sequence and prophage regions, have been shown to be superior markers for differentiating Las isolates from different geographical origins [11]–[13].

The 1.26 Mb Las genome contained at least two prophages, named FP1 and FP2, in the Las genome obtained from infected psyllid 62 [6], [13], [14] or SC1 and SC2 in the Las genome obtained from infected periwinkle [15]. Subsequently, the contribution of the prophages and phage-related gene sequences to the genetic diversity of Las was investigated in global isolates [6], [11], [13], [16]. As a result, two homologous genes, *lasA*_I_ and *lasA*_II_, (previously named as *hyv*_I_ and *hyv*_II_) were identified within the prophage FP1 and FP2, respectively in the Florida Las Psy62 genome. The *lasA*_I_ gene contained 12 nearly identical tandem repeat (NITRs) 132 bp in length and 4 partial repeats; whereas the *lasA*_II_ gene only contained one partial repeat. Because of the high copy number of the NITRs in these two genes, primers and probes were designed from the 132 bp repeat unit, and a more sensitive real-time PCR (RT-PCR) method was developed for detecting samples with low titers of Las bacteria [17]. In addition, the proteins expressed from these two genes via alternative expression systems have been characterized as novel autotransporters and were therefore renamed to *lasA*_I_ and *lasA*_II_ [18]. Further characterizations of these two genes revealed they were critical effectors in the HLB progression [19].

Genetic diversity investigations provide an understanding of the population structure and dynamics of bacteria, as well as information for determining the source of infection and their potential impact on epidemiology. Identification and genetic diversity studies on the *lasA*_I_ and *lasA*_II_ genes from Florida and other global isolates revealed not only the extensive variations in the intragenic repeat numbers, repeat arrangements, and the sequences flanking the repeat region, but also suggested multiple introductions of this bacterial pathogen into Florida [13]. In this study, we characterized the *lasA*_I_ and *lasA*_II_ genes of the Las isolates collected from infected *Citrus spp*. and *D. citri* in different geographical regions of Thailand in an effort to define the genetic relationships amongst these Las isolates.

## Materials and Methods

### Sample collection

**Figure 1:**
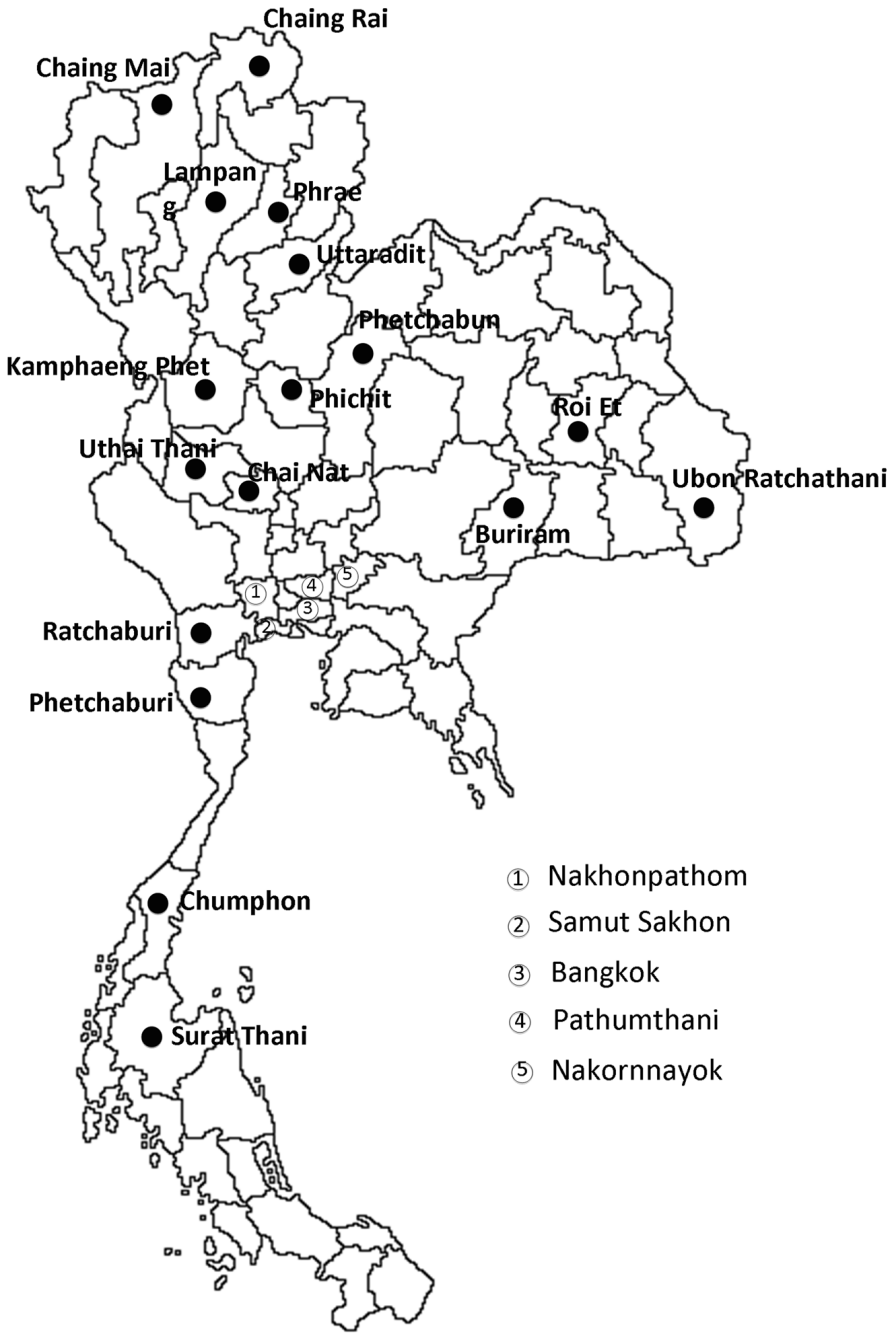
Map of sampling sites in Thailand for Las infected citrus and psyllid samples used in this study. Samples were collected from different provinces in Thailand as indicated by the black spots.

Two hundred thirty-nine symptomatic citrus leaf samples and 55 psyllids were collected from 239 HLB-affected citrus plants in different orchards throughout 22 provinces in Thailand from September 2010 to December 2012 (Figure 1). In this study, both citrus and psyllid samples were collected from 5 different citrus varieties: mandarin (*Citrus reticulata*), sweet orange (*C. sinensis*), pummelo (*C. maxima*), lime (*C. aurantifolia*), and Kaffir lime (*C. hystrix)*. Most of these varieties are commercial citrus species in Thailand.

### DNA extractions

Total DNA of each plant sample was prepared from 0.2 g of Las-infected leaf midribs. The midribs were frozen in liquid nitrogen and quickly ground to fine powder with a mortar and pestle. The leaf powder was transferred to a 1.5 mL centrifuge tube and total DNA was extracted using a DNeasy Plant Mini Kit (Qiagen,Valencia, CA, USA) according to the manufacturer's instructions. The DNA was eluted with 100 µL of distilled water and kept in -20°C until assayed. Individual insect DNA was extracted according to the method previously reported [20]. Briefly, a single psyllid was ground in a 2 mL screw capped tube containing 5 glass beads and 500 µL extraction buffer (0.1 M NaCl, 0.2 M sucrose, 50 mM EDTA, 50 mM Tris-HCl pH 8.0, and 1.25% SDS) using FastPrep-24 Homogenizer (MP Biomedicals, LLC, OH USA). Ground samples were incubated at 65°C for 15 minutes followed by the addition of 160 µL of solution III (5 M potassium acetate, 3 M glacial acetic acid), and 500 µL of 24∶1 chloroform-isoamyl alcohol mixture. After being mixed by inversion, the samples were placed on ice for 10 minutes before centrifugation at 14,000 rpm for 10 minutes. The aqueous phase was transferred to a new 1.5 mL tube and the DNA was precipitated by using an equal volume of isopropanol. The DNA pellets were washed twice with 75% ethanol and dried using a speed vacuum. The DNA pellets were then resuspended in 40 µL of distilled water and kept at −20°C until assayed.

### Detection of *Ca*. Liberibacter asiaticus by Real Time PCR

To evaluate the titer of Las bacteria in plant and psyllid DNA, the extracted DNA was initially analyzed by real-time PCR using primers and probes targeting the 16s rRNA gene and the intergenic tandem-repeat regions of *lasA*_I_ and *lasA*_II_ gene in Las genome. TaqMan real-time PCR amplification was performed in a Master Cycler Realplex Real-Time PCR System (Eppendorf Inc., USA) using specific primers HLBasf, HLBr and probe HLBp as listed in Table 1. The 15 µL TaqMan PCR reaction mixture contained 7.5 µL of TaqMan PCR master mix (Applied Biosystems), 250 nM of each primer, 150 nM of probe, and 100 ng of DNA template. The real-time PCR amplification setting included 95°C denaturation for 5 minutes, followed by 40 cycles of 94°C for 3 seconds and 60°C for 30 seconds. SYBR Green1 real-time PCR was used to detect amplicons produced with primer set LJ900f/r (Table 1), which targets the repeat region of *lasA*_I_ and *lasA*_II_. The 15 µL reaction mixture contained 7.5 µL of SYBR Green master mix, 600 and 900 nM of LJ900f and LJ900r respectively, and 100 ng of DNA template. The thermal cycling conditions were as follows: 95°C denaturation for 3 minutes followed by 40 cycles at 95°C for 3 seconds, and 62°C for 30 seconds, with fluorescence signal capture at the end of each 62°C step followed by a default melt (dissociation) stage.

**Table 1:** Primers and probes used in this study.

Name	Primer	Target gene	Reference
**Clone Amplification**			
M13f	5′GTAAAACGACGGCCAG3′	Insert amplification	
M13r	5′CAGGAAACAGCTATGAC3′	Insert amplification	
**Real-Time PCR**			
HLBasf	5′TCGAGCGCGTATGCGAATACG3′	16S rRNA	[36]
HLBasp	5′GCGTTATCCCGTAGAAAAAGGTAG3	16S rRNA	[36]
HLBasr	5′AGACGGGTGAGTAACGCG3′	16S rRNA	[36]
LJ900f	5′GCCGTTTTAACACAAAAGATGAATATC3′	*lasA*_I_/*lasA*_II_	[17]
LJ900r	5′ATAAATCAATTTGTTCTAGTTTACGAC3′	*lasA*_I_/*lasA*_II_	[17]
**Conventional PCR**			
CGO3f	5′RGGGAAAGATTTTATTGGAG3′	16S rRNA	[21]
CGO5r	5′GAAAATAYCATCTCTGATATCGT3′	16S rRNA	[21]
LJ730	5′TTGCGACTAAAGACAACGAG3′	5′ Flanking region of *lasA*_I_	[13]
LJ729	5′TTGCTAGTCTTATCGGCTTATC3′	3′ Flanking region of *lasA*_I_	[13]
LJ812	5′CCACGGAATACATCAAAGCTC3′	5′ Flanking region of *lasA*_II_	[13]
LJ1089	5′TTAGTCATCAAAATTATTAAC3′	*lasA*_II_ gene	[13]

### Conventional PCR

All primers for conventional PCR are listed in Table 1. Las-infected samples were confirmed using the CGO3F/CGO5R primer pair targeting 16S rRNA [21]. DNA samples that tested Las positive by RT-PCR were used for amplification of *lasA*_I_ and *lasA*_II_ genes. The primer pair LJ730/LJ729 were used to amplify the fragment including the *lasA*_I_ full gene and its flanking region; the *lasA*_II_ full gene and its flanking regions were amplified by the primer pair LJ812/LJ1089. The PCR reaction was performed in a 20 µL reaction mixture containing 10 µL of 2x buffer D (Epicentre Biotechnology, Madison, WI, USA), 250 nM forward/reverse primers, 1.25 U *Taq*-DNA polymerase (New England BioLads Inc., Ipswich, MA, USA) and 1–2 µL of template DNA. The PCR cycles were initiated with 95°C for 3 minutes, follow by 40 cycles of 94°C for 20 seconds, 50–54°C for 30 seconds, 72°C for 2 to 3 minutes according to different primer sets, and the final extension at 72°C for 10 minutes. The amplified PCR products were separated by electrophoresis in 1% agarose gels (1x TAE buffer) containing ethidium bromide (0.5 µL/mL) and photographed under an UV illuminator.

### Cloning and sequencing

Conventional PCR products were ligated into TOPO TA vector pCR2.1 and the *Escherichia coli* TOP10 chemical competent cells were transformed with the ligation products using TOPO TA vector pCR2.1 cloning kits (Life Technologies, Carlsbad, CA) according to the manufacturer's instructions. White colonies were selected and analyzed for positive clones via colony PCR using vector based primers M13f and M13r (Table 1). Recombinant clones were grown in Luria Bertani broth containing 50 µg/mL kanamycin and incubated overnight at 37°C with agitation. The plasmid DNA was extracted using the NucleoSpin Plasmid Quick Pure Kit (Macherey-Nagel, Germany) and diluted to 100 ng/µL with sterilized distilled water before sequencing. DNA sequencing was performed in the U.S. Horticultural Research Laboratory Core Genomics Facility using BigDye Terminator version 3.1 and the 3730×1 DNA analyzer (Applied Biosystems). Sequences were assembled by ContigExpress using Vector NTI (Life Technologies) and analyzed by Align X in Vector NTI for sequence alignment study. ORFs were predicted from *lasA*_I_ and *lasA*_II_ gene sequence using SEQtools version 8.3 (Soeren W. Rasmussen, Denmark http://www.seqtools.dk/).

### Sequence and phylogenetic analysis

BLAST program was used to compare the similarity between the newly obtained DNA sequences with the homologous sequences published in the NCBI GenBank. Phylogenetic relationships for protein sequences were inferred using parsimony and maximum likelihood approaches. For each protein, a multiple alignment was produced in Mesquite v.2.73 using ClustalW v.2.0.12 with default settings [22], [23], followed by manual adjustments. This data was analyzed in ProtTest v.2.4 with Akaike Information Criterion, resulting in a best fit substitution model for amino acid replacement (JTT+G+F; [24]–[26]). Maximum likelihood inference was conducted using RaxML v7.2.6 on the CIPRES teragrid portal with default settings and JTT, followed by 1000 bootstrap replicates [27]. These 1000 trees were used to construct a majority rule consensus tree in PAUP. Using maximum parsimony, a heuristic search with random stepwise addition and tree bisection-reconnection was implemented in PAUP. Support was assessed using NJ bootstrap (1000 replicates).

## Results

### Las population dynamics in infected citrus plants and psyllids in Thailand

A total of 239 DNA samples extracted from Las-infected citrus leaves of different geographical origins collected throughout Thailand were used in this study (Figure 1). The 239 citrus samples were collected from different varieties of citrus plants with HLB symptoms including leaf mottling, yellowing or nutritional deficiency-like symptoms. In addition, 55 psyllid samples were collected from the infected citrus plants in the same area. Las bacteria titers in the 239 citrus and 55 psyllid samples were determined by 16S rDNA-based RT-PCR with a cycle threshold (Ct) value between 14 to 35 (data not shown), with the average Ct value for the 239 infected citrus and 55 infected psyllid DNA sample being 23.31 and 20.57, respectively. Further confirmation of the presence of Las was performed by conventional PCR using primer set CGO3f/CGO5r (Table 1) to amplify a 534 bp amplicon of 16S rDNA (data not shown).

In this study, evaluation of the genetic diversity of the Las positive Thai isolates was based on two homologous tandem repeat genes, *lasA*_I_ and *lasA*_II,_ which are found within the prophage region sequences FP1 (prophage 1) and FP2 (prophage 2), respectively. RT-PCR primers LJ900f/r (Table 1), which target an identical region found in both the *lasA*_I_ and *lasA*_II_ genes [17], were used initially to investigate the presence/absence of the Las prophage sequence in the Thai isolates. All 239 citrus samples and 55 psyllid samples tested positive by RT-PCR using LJ900f/r primers with Ct values from 14–30, indicating the presence of *lasA*_I_ and/or *lasA*_II_ genes in all Las positive Thai isolates.

**Figure 2:**
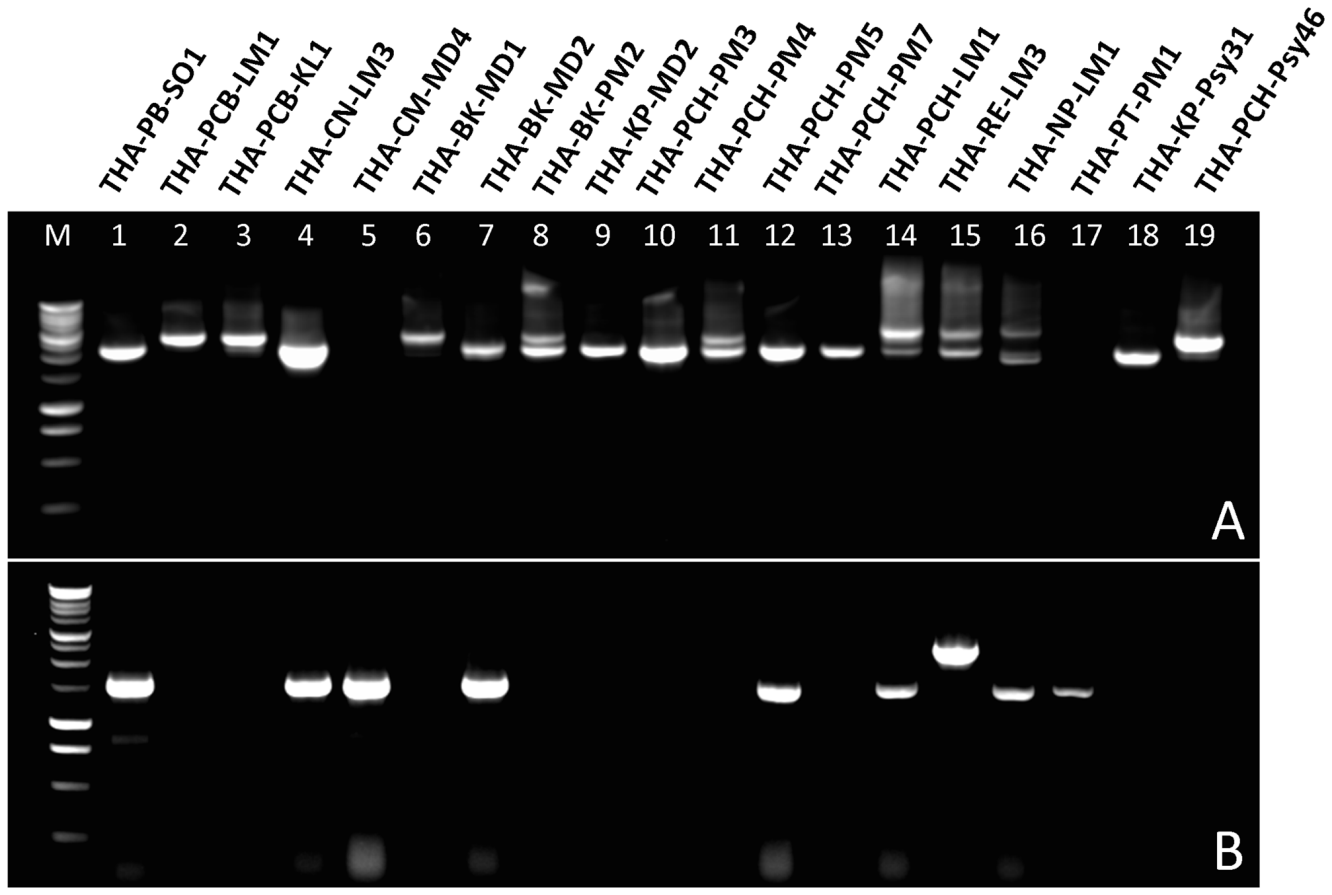
PCR amplification of *lasA*_I_ (A) and *lasA*_II_ (B) gene and flanking region from Las-infected citrus and psyllid samples from Thailand. M: 1-kb DNA ladder (Promega), lane 1–19: DNA from Las- infected *Citrus sinensis* in Phetchabun province (lane 1), *Citrus aurantifolia, Citrus hystrix* in Phetchaburi province (lane 2–3), *Citrus aurantifolia* in Chai Nat province (lane 4), *Citrus reticulata* in Chiang Mai province (lane 5), *Citrus reticulata* (lane 6–7) and *Citrus maxima* (lane 8) in Bangkok*; Citrus reticulata* in Kamphaeng Phet province (lane 9); *Citrus maxima* (lane 10–13) and *Citrus aurantifolia* (lane14) in Phichit province; *Citrus aurantifolia* in Roi Et province (lane 15); *Citrus aurantifolia* in Nakhon Pathom province (lane 16); *Citrus reticulata* in Pathumthani province (lane 17); *Diaphorina citri* in Kamphaeng Phet province (lane 18) and Phichit province (lane 19).

Further in depth evaluation of the genetic diversity within the Thai isolates was conducted using conventional PCR to individually amplify the *lasA*_I_ and *lasA*_II_ genes and their flanking regions from all 239 infected citrus and 55 psyllid DNA samples using primer set LJ729/LJ730 and LJ812/LJ1089 as described previously (Table 1). The representative PCR results from the different provinces are shown in Figure 2. The different size and/or multiple sized amplicons observed in the agarose gel reveal the genetic diversity of the prophage region sequences amongst the Thai isolates. The dynamics of the Las prophage populations in the different citrus varieties and psyllids collected from Thailand are indicated in Table 2. Of the 294 Las positive DNA samples from Thailand, 142 (48.29%) contained *lasA*_I_, 186 (63.26%) contained *lasA*_II_, and 57 (19.38%) contained both *lasA*_I_ and *lasA*_II_. Interestingly, more Las isolates from HLB-affected citrus plants contained *lasA*_II_ (71.12%) than *lasA*_I_ (37.23%). In contrast, Las isolates from infected psyllids contained *lasA*_I_ more frequently (96.36%) than *lasA*_II_ (29.09%).

**Table 2:** Evaluation of the Las population in different hosts collected from different geographical locations in Thailand based on two hyper variable tandem repeat genes in the prophage region of the Las genome.

		Number of positive			
Host	HLBaspr/LJ900^a^	*lasA*_I_ b	*lasA*_II_ b	*lasA*_I_ and *lasA*_II_ c	*ΔlasA*_I_ and *ΔlasA*_II_ d
*Citrus sinensis*	11	5(45.45%)	8(72.72%)	2(18.18%)	0(0%)
*Citrus aurantifolia*	78	24(30.76%)	64(82.05%)	17(21.79%)	7(8.97%)
*Citrus reticulata*	53	21(39.62%)	39(73.58%)	13(24.52%)	6(11.32%)
*Citrus maxima*	61	25(40.98%)	35(57.37%)	7(11.47%)	8(13.11%)
*Citrus hystrix*	36	14(38.88%)	24(66.66%)	4(11.11%)	2(5.55%)
**Total (Citrus)**	**239**	**89(37.23%)**	**170(71.12%)**	**43(17.99%)**	**23(9.62%)**
*Diaphorina citri*	55	53(96.36%)	16(29.09%)	14(25.45%)	0(0%)
**Total (Citrus + Psyllid)**	**294**	**142(48.29%)**	**186(63.26%)**	**57(19.38%)**	**23(7.82%)**

a Number of Las positive samples was determined by *TaqMan* real-time PCR (Ct values between 14–35) and SYBR GREEN 1 real-time PCR (Ct values between 14–30) using16S rDNA based primers/probe [36] and LJ900 primers targeting the *lasA*_I_ and *lasA*_II_ repeat sequence [17], respectively.

b Conventional PCR results using primers targeting *lasA*_I_ or *lasA*_II_, respectively.

c Number and percentage of samples determined to be positive for both *lasA*_I_ and *lasA*_II_.

d Number and percentage of samples determined to be negative for both *lasA*_I_ and *lasA*_II_.

Of note is the fact that 23 samples (7.82%) were undetectable using both the *lasA*_I_ and *lasA*_II_ primers in conventional PCR despite an average Ct value of 21.65 for the 16S rDNA using RT-PCR (data not shown) and a positive result using the LJ900f/r primer pair. Among these 23 samples, 10 contained a low titer of *lasA*_I_ or *lasA*_II_ with a Ct value ranging from 25–30 by SYBR Green real-time PCR using primers LJ900f/r, which may explain why the 10 samples tested negative by conventional PCR using both *lasA*_I_ and *lasA*_II_ region primers. However, 13 contained a higher titer of *lasA*_I_ or *lasA*_II_ with a Ct value from 19–24 by LJ900f/r, thus eliminating low titer as a reason why the *lasA*_I_ and *lasA*_II_ primers could not amplify in these samples.

The association of the Las genetic diversity with regard to their geographical origins in Thailand was also investigated in this study (Table 3). Except for the samples from Lampang, Bangkok, Nakornnayok, Chai Nat, Kamphaeng Phet and Phichit province, Las isolates from other provinces contained more *lasA*_II_ than *lasA*_I_. Furthermore, all samples from 4 provinces (Chiang Rai, Pathum Thani, Ubon Ratchathani and Buriram) were positive for *lasA*_II_ only.

**Table 3:** Evaluation of Las citrus isolates from different geographical locations in Thailand using primers targeting the *lasA*_I_ and *lasA*_II_ gene regions within the prophage.

		Number of positive			
Geographical origin	HLBaspr/LJ900^a^	*lasA*_I_ b	*lasA*_II_ b	*lasA*_I_ and *lasA*_II_ c	*ΔlasA*_I_ and *ΔlasA*_II_ d
Chiang Mai	11	5(45.45%)	9(81.81%)	3(27.27%)	0(0%)
Chiang Rai	7	0(0%)	7(100%)	0(0%)	0(0%)
Phrae	6	1(16.66%)	2(33.33%)	0(0%)	3(50%)
Uttaradit	5	2(40%)	4(80%)	1(20%)	0(0%)
Lampang	2	1(50%)	1(50%)	0(0%)	0(0%)
Bangkok	33	24(72.7%)	16(48.48%)	10(30.305%)	3(9.09%)
Samut Sakhon	6	3(50%)	4(66.66%)	1(16.66%)	0(0%)
Nakhon Pathom	22	8 (36.36%)	18(81.82%)	4(18.18%)	1(4.55%)
Pathum Thani	7	0(0%)	1(14.28%)	0(0%)	6(85.71%)
Nakornnayok	6	5(83.33%)	1(16.66%)	1(16.66%)	1(16.66%)
Uthai Thani	7	1(14.28%)	7(100%)	1(14.28%)	0(0%)
Chai Nat	13	6(46.15%)	6(46.15%)	3(23.07%)	3(23.07%)
Phetchabun	7	1(14.28%)	7(100%)	1(14.28%)	0(0%)
Kamphaeng Phet	11	6(54.54%)	5(45.45%)	2(18.18%)	2(18.18%)
Phichit	18	11(61.11%)	9(50%)	5(27.78%)	3(16.67%)
Ubon Ratchathani	3	0(0%)	3(100%)	0(0%)	0(0%)
Buriram	3	0(0%)	3(100%)	0(%)	0(0%)
Roi Et	10	3(30%)	8(80%)	2(20%)	1(10%)
Phetchaburi	29	4(13.79%)	26(89.65%)	1(3.44%)	0(0%)
Ratchaburi	11	3(27.27%)	11(100%)	3(27.27%)	0(0%)
Chumphon	7	4(57.14%)	7(100%)	4(57.14%)	0(0%)
Surat thani	6	1(16.66%)	6(100%)	1(16.66%)	0(0%)
Total	239	89(37.23%)	170(71.12%)	43(17.99%)	23(7.82%)

a Number of Las positive sample was determined by *TaqMan* real-time PCR (Ct values between 14–35) and SYBR GREEN 1 real-time PCR (Ct values between 14–30) using16S rDNA based primers/probe [36] and LJ900 primers targeting the *lasA*_I_ and *lasA*_II_ repeat sequence [17], respectively.

b Conventional PCR results using primers targeting *lasA*_I_ or *lasA*_II_, respectively.

c Number and percentage of samples determined to be positive for both *lasA*_I_ and *lasA*_II_.

d Number and percentage of samples determined to be negative for both *lasA*_I_ and *lasA*_II_.

### Variations of *lasA*_I_ and *lasA*_II_ in Las-infected citrus plants and psyllids in Thailand

**Figure 3:**
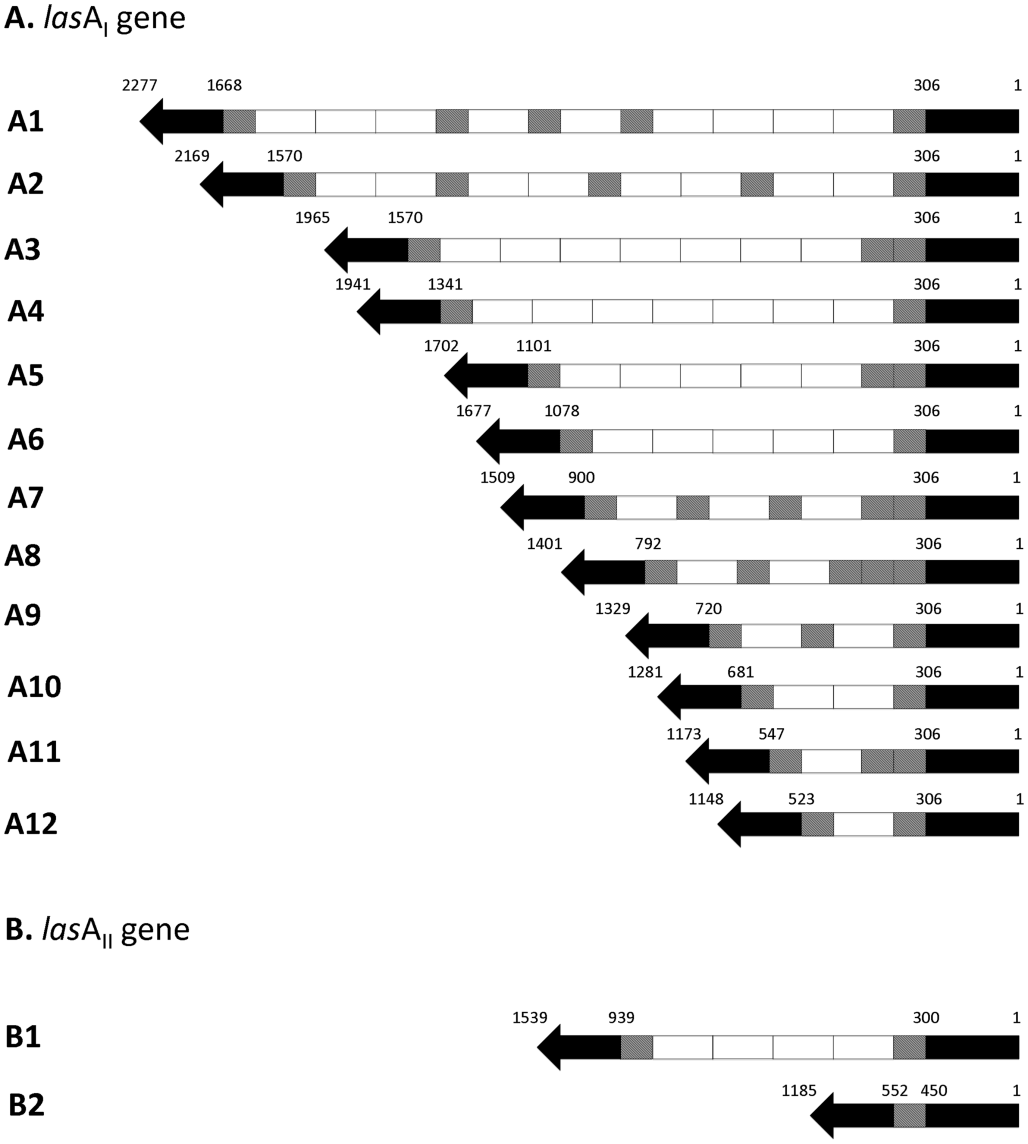
Schematic diagram presenting the full/partial repeat numbers and arrangement in *lasA*_I_ (A) and *lasA*_II_ (B) from Las Thai isolates. The arrows in A and B denote the open reading frames (ORF) of *lasA*_I_ and *lasA*_II_. The number of repeat units within each ORF is indicated with each light grey box representing a full 132 bp tandem repeat unit and each dark grey box representing a partial repeat unit. The numbers above the *lasA*_I_ and *lasA*_II_ genes indicates the gene size and the position of the repeat region in the *lasA*_I_ and *lasA*_II_ gene.

A total of 271 of the 294 Las positive citrus and psyllid DNA samples amplified *lasA*_I_ and/or *lasA*_II_ genes using primer pairs LJ730/LJ729 and LJ812/LJ1089, respectively, in conventional PCR. Twenty *lasA*_I_ and six *lasA*_II_ amplicons from different varieties of citrus or psyllids were selected for cloning and sequence analysis. The sequencing result confirmed that the presence of the different sized amplicons using the two set of primers were indeed *lasA*_I_ and *lasA*_II_ and their flanking region sequences (Table 4). Sequence alignments revealed that the samples containing multiple sized amplicons and the size variants seen amongst the different samples were due to changes in the number of repeats (full or partials) and/or the arrangement of the individual repeat units within *lasA*_I_ and *lasA*_II_. Overall, the *lasA*_I_ gene products showed more sequence variation with regards to the partial repeat than the *lasA*_II_ gene in both the citrus and psyllids samples (Figure 2 and Figure 3). The number of full repeats varied from 1 to 9 in *lasA*_I_, with a majority having 1, 2 or 7 full repeats (Table 4), while the arrangement of the full and partial repeats in *lasA*_I_ gene was highly variable as well (Figure 3A). Each full length repeat in *lasA*_I_ was 132 bp whereas the size of the partial repeats in all of the amplicons varied from 24, 33, 48, 78 and 108 bp. Commonly, a 78 bp partial repeat at the 5′ end and a 33 bp partial repeat at 3′ end of the entire intragenic repeat region were observed in each *lasA*_I_ gene, while a majority of the other sizes (24, 48 and 108 bp) of partial repeats were observed between full-length repeats inside the repeat region (Figure 3). The predicted open reading frames (ORFs) of *lasA*_I_ from Las Thai isolates separated into the following 12 distinct patterns when the ratio of full versus partial repeats was considered: 9/5, 8/5, 7/3, 7/2, 5/3, 5/2, 3/5, 2/5, 2/3, 2/2, 1/3, 1/2. However, the pattern of 7 full and 2 partial repeats was the dominant group from the 20 sequenced *lasA*_I_ amplicons (Table 4). The division of the ORFs of *lasA*_I_ into the twelve subclasses is illustrated (Figure 3A). Based on the sequence homology of the *lasA*_I_ gene, the level of identity among the Thai isolates was 87%–99%. These isolates were more closely related to China (CHA-Cit18_pLJ316.1) and the Philippines (PHA-Psy5_pLJ313.4) isolates, sharing approximately 89%–99% identity. In contrast, Florida (FL-Psy62_pLJ108.1) and India (IND-Psy2_pLJ314.1) isolates only share 86%–97% identity with Thai isolates. The 5′ end of the DNA sequences outside of the repeat region share 98–100% similarity among Thai isolates and 92–94% similarity when compared to the same sequenced region from isolates from Florida, China, the Philippines, and India. The 3′ ends of the DNA sequences outside of the repeat region were more variable than 5′ ends. The similarity between Thai isolates varied from 81–100%, and the 3′ end of the DNA sequences outside of repeat region were more closely related to the China and Philippine isolates (92–100% similarity) when compare with those from India and Florida (88–93% similarity).

**Table 4:** The *lasA*I*/lasA_II_* genes in Las Thai isolates from infected citrus and psyllids.

	*lasA*_I_ gene			*lasA*_II_ gene			
Sample name	Clone ID	No. of full/partial repeats	Accessions no. (Reference sequence)		Clone ID	No. of full/partial repeats	Accessions no. (Reference sequence)
THA-PB-SO1	pHYVI1.6	2/5		pHYVII1.4		0/1	
THA-PCB-LM1	pHYVI4.13	7/2		-			
THA-PCB-KL1	pHYVI8.1	5/2		-			
THA-CN-LM3	pHYVI12.6	7/2		NC			
THA-CM-MD2	pHYVI140.6	9/5		NC			
THA-CM-MD4	-	-		pHYVII19.1		0/1	
THA-BK-MD1	pHYVI20.1	7/3		-			
	pHYVI20.4	1/3					
THA-BK-MD2	pHYVI21.1	1/2		pHYVII21.9		0/1	
	pHYVI21.14	2/3					
THA-BK-MD6	pHYVI138.3	3/5		NC			
THA-BK-PM2	pHYVI77.9	7/2		-			
	pHYVI77.12	1/2					
THA-KP-MD2	pHYVI37.1	2/5		-			
THA-KP-Psy31	pHYVI-Psy31.24	1/2		-			
THA-PCH-PM3	pHYVI50.1	1/2		-			
	pHYVI50.4	7/2					
THA-PCH-PM4	pHYVI51.2	1/2		-			
	pHYVI51.3	2/3					
THA-PCH-PM6	pHYVI53.2	7/2		NC			
	pHYVI53.11	2/2					
THA-PCH-PM7	pHYVI54.1	2/2		-			
THA-PCH-PM8	pHYVI55.7	7/2		-			
THA-PCH-LM1	pHYVI58.1	7/2		NC			
	pHYVI58.8	1/2					
THA-PCH-Psy17	NC			pHYVII-Psy17.4		0/1	
THA-PCH-Psy42	NC			pHYVII-Psy42.1		0/1	
THA-PCH-Psy46	pHYVI-Psy46.10	7/2		-			
THA-RE-LM3	pHYVI63.1	1/3		pHYVII63.1		4/2	
	pHYVI63.9	5/3		pHYVII63.6		0/1	
THA-NP-LM1	pHYVI68.1	1/3		NC			
	pHYVI68.2	8/5					
THA-PT-PM1	-			pHYVII100.1		0/1	
FL-Psy62(MDA)*	pLJ108.1	12/4	YP_003084345.1	pLJ201.1		0/1	HQ263703
CHA-Cit18*	pLJ316.1	7/2	HQ263691	-		-	
CHA-Cit5*	-	-		pLJ393.1		1/0	HQ263715
THA-KP2.3*	pLJ342.1	7/2	HQ263693	-		-	
PHA-Psy5*	pLJ313.4	7/2	HQ263695	-		-	
IND-Psy2*	pLJ314.1	4/1	HQ263699	-		-	

‘NC’ PCR positive but not cloned,

‘-’ PCR negative by *lasA*_I_ or *lasA*_II_ primers.

*Identified by Zhou et al. (2011) [14].

Compared to *lasA*_I_, the *lasA*_II_ gene was less diversified and the sequence variations among *lasA*_II_ gene amplicons were at the single nucleotide polymorphism level. All PCR products showed a single band with the expected size of the amplicon being approximately 1600 bp using the LJ812/LJ1089 primers. Most *lasA*_II_ amplicons were of the expected size (Figure 2B) despite being amplified from different samples collected from different geographic regions. The ORF of the *lasA*_II_ gene from the Thai isolates contained only 2 patterns with a ratio of full to partial repeats of 4/2 or 0/1 (Figure 3B, B1–B2).The dominant pattern present was 0 full and 1 partial repeat and was observed in 6 *lasA*_II_ amplicons (Table 4). These *lasA*_II_ sequences, isolated from both citrus and psyllids samples, shared 99% identity with the repeat sequence from China (CHA-Cit5_pLJ393.1) suggesting that the *lasA*_II_ gene found in Las strains from Thailand possess little variation and are closely related to isolates from China. In contrast, only one clone from the THA-RE-LM3 isolate collected from Roi Et province (*Citrus aurantifolia)* contained 4 full repeat sequences and one partial repeat and was 95% identical to the Florida isolate (FL-Psy62_pLJ201.1) (Figure 3B, B1).

ORF prediction for the *lasA*_I_ and *lasA*_II_ gene sequences from the 26 amplicons indicated that most repeat regions were found to be in frame when the genes were translated using SEQtools 8.4 software. Therefore, deletion or insertion of the full or partial repeat unit did not disrupt the open reading frame of *lasA*_I_ or *lasA*_II_ genes but merely changed the length of the expected protein product.

### Diversity and Phylogenetic analysis of *Ca*. Liberibacter asiaticus in Thailand

**Figure 4:**
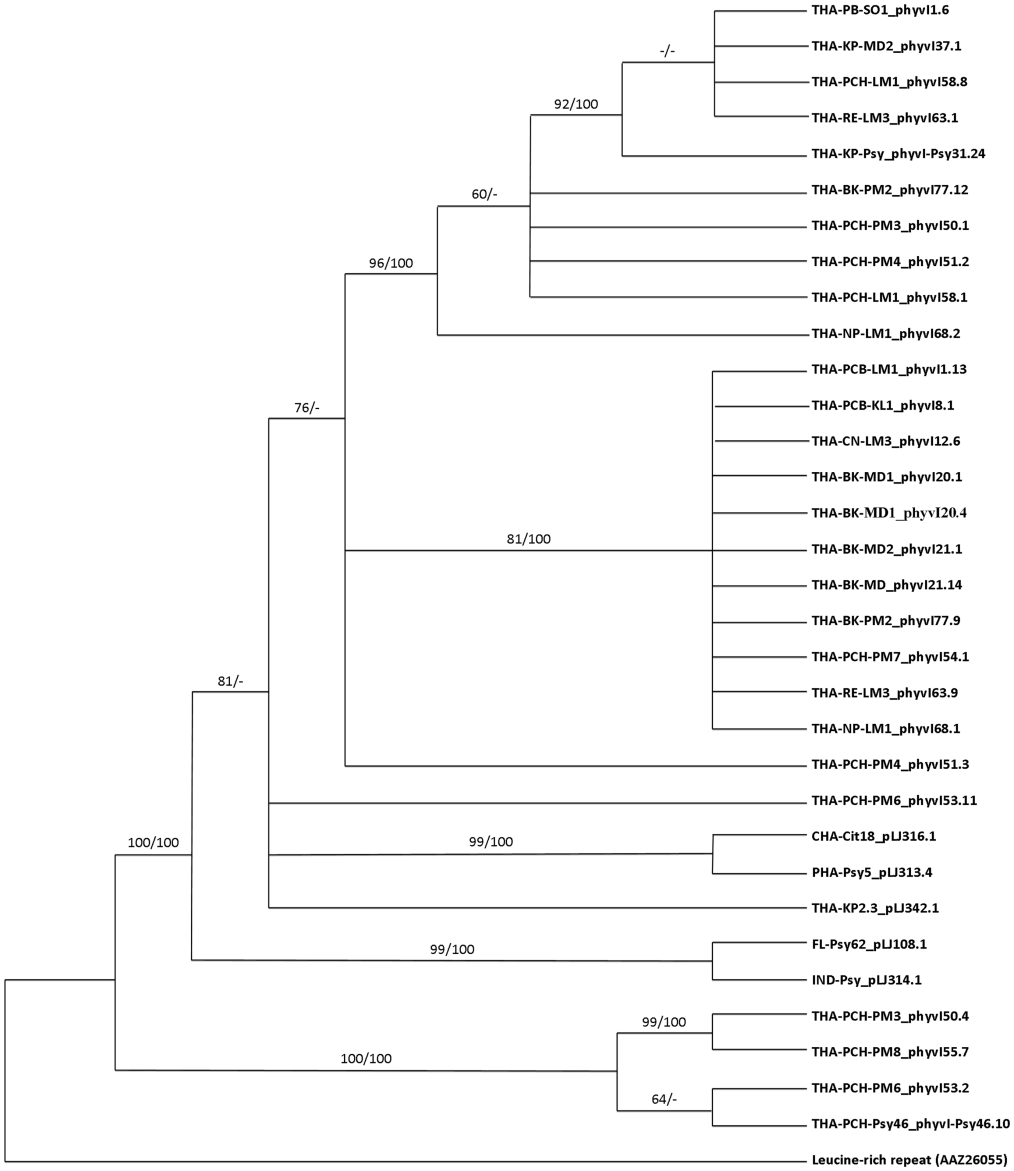
Majority rule consensus tree based on maximum likelihood analyses of 33 amino acid sequences representing *lasA*_I_ gene (with leucine-rich repeat protein from *Colwellia psychrerythraea* as the outgroup). Branch support values represent RaxML (≥60%) bootstrap before the slash and maximum-parsimony bootstrap (≥60%) values after the slash. Values below 60% are indicated by an (−).

**Figure 5:**
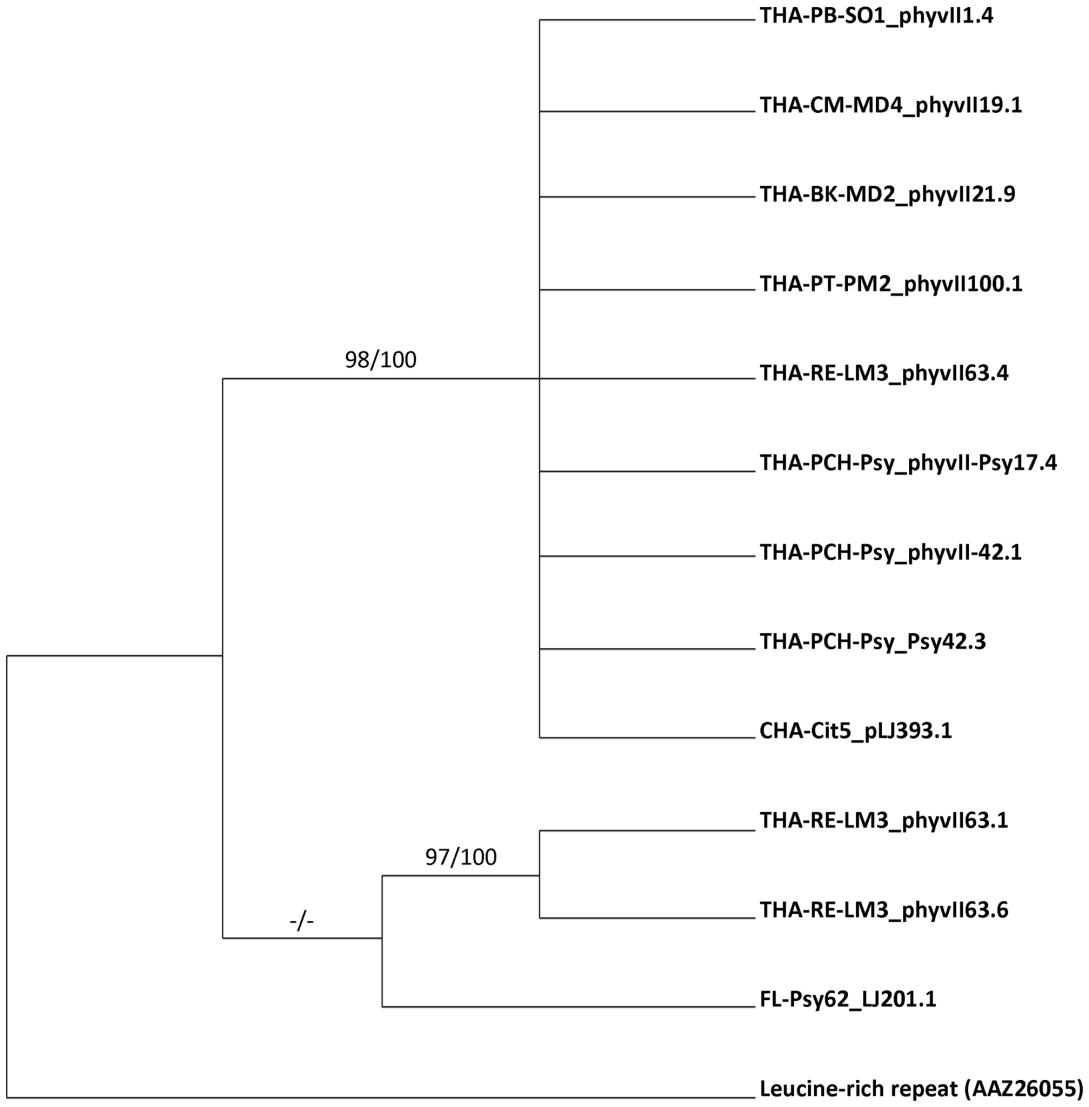
Majority rule consensus tree based on maximum likelihood analyses of 13 amino acid sequences representing the *lasA*_II_ gene (with leucine-rich repeat protein from *Colwellia psychrerythraea* as outgroup). Branch support values represent RaxML (≥60%) bootstrap before the slash and maximum-parsimony bootstrap (≥60%) after the slash. Values below 60% are indicated by an (−).

The *lasA*_I_ and *lasA*_II_ gene sequences from the Thai Las isolates were deposited to GenBank. Sequence alignments with *Ca*. L. asiaticus str. psy62 (Accession # NC_012985) and China (Accession # NC_020549) were subsequently performed. Based on the variable nucleotide sequences of Las isolates from citrus and psyllid samples collected from different locations in Thailand, DNA and amino acid sequences deduced from the *lasA*_I_ gene and the *lasA*_II_ genes were aligned and integrated into phylogenetic analyses (Figure 4 and 5). Previously identified LasA_I_ or LasA_II_ protein sequences from Las isolates in Florida (YP_0033084345.1, HQ263703), China (HQ263691, HQ263715), the Philippines (HQ263695), India (HQ263699) and Thailand (HQ263693) were included in these phylogenetic analyses. The leucine-rich repeat protein homolog from *Colwellia psychrerythraea* (Accession # AAZ26055) was used as the outgroup for both LasA proteins. Phylogenetic analyses of the *lasA*_I_ gene using the maximum parsimony yielded 680 best trees with tree lengths of 1095 steps. Bootstrap support ≥60% was observed for the 12 nodes. The *lasA*_I_ gene yielded only 6 trees with tree lengths of 581 steps. Bootstrap support ≥60% was observed for the 2 nodes. A comparison of the tandem repeat region of the *lasA*_I_ gene from Thai isolates with other worldwide samples revealed that most of the Thai Las samples formed a well-supported sister-clade with China (CHA-Cit18_pLJ316.1) and Philippine isolates (PHA-Psy5_pLJ313.4). The Florida (FL-Psy62_pLJ108.1) and India (IND-Psy2_pLJ314.1) isolates formed a well-supported sister-clade (100% bootstrap) to this group. The *lasA*_I_ gene from 4 isolates, collected from pummelo and psyllid in the Phichit province, grouped together and demonstrated a branch support of 100% bootstrap. LasA_II_ phylogenetic inferences, containing 13 taxa, resulted in a tree with a well-supported sister-clade with 98% bootstrap to the sample from China (CHA-Cit5-pLJ393.1). Two *lasA*_I_ genes from Roi Et province isolates formed a well-supported sister-clade to Florida psyllid (FL-Psy-pLJ201.10) although without bootstrap support.

## Discussion

*Ca.* Liberibacter asiaticus encodes two intriguing autotransporters, LasA_I_ and LasA_II_ [18], which were used to evaluate the Las population diversity among Florida and international isolates [13]. In this study, the same primers were utilized to investigate the Las populations in infected citrus and psyllids from different geographical locations in Thailand. The PCR and sequencing results of these two hyper variable regions (*lasA*_I_/*lasA*_II_) confirmed the presence of the homologous FP1 and/or FP2 prophages in Las Thai isolates (Table 2 and 3), and validated that the protocol is sufficient for the characterization and differentiation of Las populations among isolates in Thailand. Based on the previous study, most if not all Florida Las isolates contained both *lasA*_I_ and *lasA*_II_, which correspond to the FP1 and FP2 prophages [6]. Although the number of Las positive samples were limited in that study, all DNA extracts from samples outside of America detected either *lasA*_I_ or *lasA*_II_ as describe by Zhou [6]. With an increased sample number from Thailand, most Las-infected citrus and psyllids were found to contain either FP1 or FP2, while 17.99% of the Las-infected citrus and 25.45% of the psyllids samples contained both prophages in the Thai isolates. Interestingly, 13 citrus samples containing a high titer of Las bacteria (tested by 16s rDNA based TagMan RT-PCR) and a high copy number of *lasA*_I_ repeats (tested by *lasA*_I_ and *lasA*_II_ gene based SYBR green RT-PCR) still tested negative by conventional PCR using both *lasA*_I_ and *lasA*_II_ gene region primers. Since bacterial titers at this threshold are normally detectable by conventional PCR, we speculate the lack of a PCR product may be the result of a dramatic sequence variation in *lasA*_I_ and *lasA*_II_ and its flanking region, especially in the primer sequence's target region, which suggested the possibility of producing new prophage recombinants within the Las genome. This parallels what was seen in the Las genome from Florida [13].

Many *lasA*_I_ amplicons showed multiple bands but only a single band was ever detected for the *lasA*_II_ amplicon. This observation was consistent with that found in Florida isolates [13]. Although gene duplication within a single strain cannot be ruled out, the high variation of the *lasA*_I_ gene from the same plant sample is thought to be indicative of a co-infection by multiple populations of *Ca.* L. asiaticus in a single source plant. A majority of the amplicons of the *lasA*_I_ gene found in Thailand isolates (20 in total) contained 7 full and 2 partial repeats (Table 4), a pattern consistent with both the China isolate (CHA-Cit18, HQ263691) and the Philippines isolate (PHA-Psy5, HQ263695). However, a majority of the *lasA*_II_ genes found in Thailand isolates contained 0 full and 1 partial repeat (Table 4), a pattern opposite to the China isolate (CHA-Cit5, HQ263715), which contains 1 full and 0 partial repeats. Interestingly, only one isolate, THA-RE-LM3, contained 4 full and 2 partial repeats (Figure 3B, entry B1). This isolate shared 95% identity with the Florida isolate (HQ263703). Our LasA_II_ phylogenetic tree results agreed nicely with those previously reported by Zhou in 2011, which indicated that another major route of HLB pathogen introduction may have occurred from Thailand to Florida. Our results imply that the Las bacteria in Thailand may have been introduced from both China and the Philippines.

Historically, HLB disease, also known as yellow shoot, was first reported in southern China in 1919 [28] and the disease occurred in 1921 in the Philippines presenting as zinc deficiency symptoms [29]. HLB did not appear in Thailand until the 1960s [2], [3]. Since budwood of several mandarins was introduced into Thailand from China at the same time, it was speculated that this might be the source of Las introduction [1], [30]. Alternatively, transmissions of HLB bacteria might have resulted from the insect vector (*Diaphorina citri*) migrating from China since the psyllids found in Thailand are of the same haplotype as a majority of the psyllids in China based on the analysis of the mitochondrial cytochrome oxidase I [31].

In 1998, Ohtsu used the 16S rDNA and 16S/23S intergenic regions to evaluate genetic diversity in Thailand. The sequencing data of these regions indicated that the Nepalese and Thai isolates were closely related and were slightly different from the India and Chinese isolate [32]. Based on the prophage region, Tomimura performed a phylogenetic analysis using bacteriophage–type DNA polymerase sequences. His results revealed three clusters in Southeast Asia where Thai isolates were grouped with Vietnam isolates [12]. However, multilocus microsatellite analysis of *Ca*. L. asiaticus indicated that Thai isolates grouped with samples from Brazil and East to Southeast Asia [33]. Results from the phylogenetic analysis of *lasA*_I_ and *lasA*_II_ sequences in this study also grouped the Las Thai isolates with the Chinese isolate, providing additional evidence for a possible introduction of *Ca*. L. asiaticus into Thailand from China. The phylogenetic analysis here indicated differences in the tandem repeat numbers and protein sequences among isolates of different geographical origins although the differences in citrus cultivars and organisms (plant versus psyllid) did not appear to influence phylogenetic placement.

Although the HLB bacteria are vectored by the psyllid, the prophage populations between psyllids and citrus appear different since 96.36% of Las-infected psyllid samples contained FP1 compared to 37.23% of Las-infected citrus samples while 29.09% of Las-infected psyllid samples contained FP2 compared to 71.12% of Las-infected citrus samples. These results are consistent with previous studies when the two hyper-variable regions were used by Zhuo et al. [6] to investigate the prophage population dynamics. In that study, new prophage variants/types were identified from the FP1 and FP2 prophages. Type A was most abundant in Las-infected psyllids and is located in FP1, indicating that FP1 was the dominant population in Las-infected psyllids, which may be important for insect transmission.

Although Zhang et al. [15] described two largely homologous DNA sequences as the prophage region SC1/SC2 of Las-infected UF506 isolates, no discussion regarding the function of either prophage, their role in the virulence of the HLB disease, or disease transmission was provided. Many bacteriophage or prophage from bacterial pathogens encode virulence factors such as bacterial toxins, effectors, attachment proteins, and extracellular polysaccharides [34]. We hypothesize that the prophage infecting Las can change avirulent strains into virulent strains, similar to what is seen with other bacteria. For example, various temperate phages of the *Myovirus* and *Inovirus* families were able to infect *Ralstonia solanacearum*. Infection of *Ralstonia* by phage *φ*RSS1 alters its pathogenicity by increasing the virulence of *Ralstonia* in host plants. Prophage genes have also been predicted to encode for pathogenicity or virulence factors such as with *Xylella fastidiosa*, a pathogen of grapevines and citrus. This organism contains the XfP1 prophage, which carries a gene similar to *vapD*, a putative virulence-associated protein from the sheep pathogen *Dichelobacter* [35]. Although the function of the *lasA*_I_ and *lasA*_II_ genes has not been completely elucidated, their similarity with other known autotransporters and translocation to the mitochondria of the host cell [18] makes them likely candidates as proteins encoded by the phage that are involved in the virulence of the Las pathogen.

Finally, this is the first study to elucidate the genetic variation of the *Ca*. L. asiaticus populations in Thailand using the two hypervariable effector genes, *lasA*_I_ and *lasA*_II_, as molecular markers. Hypervariations in the *lasA*_I_ and *lasA*_II_ genes among *Ca*. L. asiaticus isolates implies a potential important mechanism about the adaptation ability of *Ca.* L. asiaticus on insect vs. plant hosts. Elucidation of the population structure, ecology, and epidemiology of the pathogen in relation to its genetic diversity and geographical location is an important step towards further effective disease management.
